# Label-free multiphoton microscopy for intraoperative identification of glioma features and tumor heterogeneity

**DOI:** 10.3389/fonc.2026.1802324

**Published:** 2026-06-22

**Authors:** León V. Duncker, Sven Richter, Roberta Galli, Matthias Meinhardt, Leon C. Hoffmann, Katrin Kirsche, Lidia Großer, Achim Temme, Ilker Y. Eyüpoglu, Ortrud Uckermann

**Affiliations:** 1 Department of Neurosurgery, Faculty of Medicine and University Hospital Carl Gustav Carus, TU Dresden, Dresden, Germany; 2 Else Kröner Fresenius Center for Digital Health, Faculty of Medicine, TU Dresden, Dresden, Germany; 3 Medical Physics and Biomedical Engineering, Faculty of Medicine, TU Dresden, Dresden, Germany; 4 Department of Pathology, Faculty of Medicine and University Hospital Carl Gustav Carus, TU Dresden, Dresden, Germany

**Keywords:** autofluorescence, brain tumor, clinical score, coherent anti-Stokes Raman sattering, intraoperative, second harmonic generation

## Abstract

**Introduction:**

Diffuse gliomas comprise a heterogeneous group of primary brain tumors with different biological behavior and aggressiveness. Intraoperative knowledge of tumor subtype and grade could enable adaptation of the surgical strategy. In this context, label-free multiphoton imaging modalities that allow *in situ* tissue characterization for glioma grading were investigated.

**Methods:**

A multimodal imaging approach combining coherent anti-Stokes Raman scattering (CARS), autofluorescence (AF), and second harmonic generation (SHG) was applied to acquire an extensive dataset from 102 glioma patients, including glioblastoma (WHO grade 4), astrocytoma (WHO grades 2, 3, and 4), and oligodendroglioma (WHO grades 2 and 3). The prevalence of specific tissue features was evaluated based on visual inspection and used to build a score for tumor malignancy.

**Results:**

CARS detected lipid droplets, AF showed cellular structures, and SHG-visualized blood vessels as well as remodeling of extracellular matrix. These were identified as histological features associated with tumor malignancy and recognizable intraoperatively by the surgeon. Quantification showed statistically significant differences in feature prevalence among tumor types, particularly between WHO grade 2 and grade 4 gliomas, despite substantial variability across patients and within individual tissue samples. Integration of multiple imaging features into a numerical score based on their presence or absence yielded values indicative of malignancy, with the highest scores observed exclusively in WHO grade 4 tumors.

**Conclusion:**

Label-free multiphoton imaging shows strong potential as an intraoperative diagnostic tool, enabling surgeons to score readily identifiable tissue features and obtain enhanced real-time information on glioma malignancy.

## 1 Introduction

Adult-type diffuse gliomas encompass a diverse group of primary brain tumors, including astrocytoma and oligodendroglioma of different malignancy grades as well as glioblastoma WHO CNS grade 4, each exhibiting distinct histopathological and molecular characteristics that influence growth patterns and clinical behavior (1). First line treatment is usually surgical resection and the extent of resection is associated to survival (2–4). Therefore, intraoperative decision making must always balance resection with preservation of functional brain tissue. At this point, knowledge of glioma subtype and malignancy grade would be of utmost importance for the neurosurgeon, as those tumors differ substantially in aggressiveness (1). However, definitive diagnosis and WHO grading relies on histopathological and molecular analysis of resected tissue samples, which is inherently retrospective, and surgeons have to take decisions regarding the extent of resection based on visual, tactile, and imaging-derived cues. In this context, intraoperative imaging modalities capable of revealing tumor-associated structural and biochemical features may provide valuable guidance, supporting the identification of tumor tissue, estimation of infiltration, and adaptation of surgical strategy according to suspected tumor grade.

Tissue morphochemistry can be visualized using multiphoton microscopy without the need for labels or contrast agents. In particular, autofluorescence (AF) imaging, second and third harmonic generation (SHG and THG) as well as coherent Raman scattering, including coherent Raman anti-Stokes scattering (CARS) and stimulated Raman scattering (SRS) are used and visualize specific tissue components in great detail. Taking advantage of these technologies, either as stand-alone solutions or in combination, holds great promise for accurately distinguishing brain tumors from non-tumor brain tissue and might provide diagnostic information on brain tumor type (5).

Previous studies addressed label-free CARS-AF-SHG imaging for brain tumor delineation. Those investigated primary and secondary brain tumors and used brain tissue from autopsy and epilepsy surgery as non-tumor controls (6, 7). Moreover, the tissue architecture of hippocampus sclerosis was investigated (8). White matter is characterized by the presence of myelinated axons that produce an intense CARS signal because of the high lipid content of myelin sheaths. In gray matter, the CARS signal intensity is lower. Lipid droplets were not detected by CARS in non-tumor brain tissue. Label-based approaches confirm the absence of lipid droplets in the healthy brain (9). Cells with cytoplasmic fluorescence are mainly found in gray matter, and were identified as pyramidal neurons in the case of human hippocampus (8). Additionally, punctate autofluorescence was frequently detected in non-tumor brain (6, 8, 10). Adventitial collagen is the major source of SHG signal in the human brain and allows the visualization of blood vessels (6, 11). Hence, SHG also visualizes corpora amylacea, a structure that might occur in the aged brain but is not considered to be disease-related (12). Label-free multiphoton microscopy is often combined with machine learning or deep learning techniques to extract tumor related features, prediction of tissue type and tumor identification (13). When applied to visualize tissue structure, CARS, AF and SHG imaging allowed discerning brain tumors and non-tumor brain tissue using texture analysis in combination with discriminant analysis (7). AF imaging in combination with SHG of collagen allowed visualization of glioma samples and classification using deep learning allowed distinguishing gray and white matter as well as non-neoplastic tissue from tumor areas (14). Third harmonic generation in combination with deep learning allowed glioma recognition in comparison to non-tumor brain samples (15). SRS imaging was successfully used for obtaining molecular diagnosis of glioma regarding IDH1 mutation, 1p19q co-deletion and ATRX mutation by employing an artificial intelligence based algorithm (16). Models trained on ~4 million images allowed the prediction of degree of infiltration in ex vivo samples (17). Moreover, artificial intelligence-based methodology combining multiphoton technologies with adaptive optics, denoising and segmentation enabled to image experimental glioblastoma growth over time in the mouse model (18). However, the performance of data-driven models might be sensitive to variations in imaging conditions, tissue preparation, and patient-specific factors, raising concerns about robustness when transferring models from ex vivo datasets to *in situ* intraoperative imaging or when analyzing datasets of different centers. Moreover, the clinical translation of AI-based diagnostic tools is further complicated by challenges related to model interpretability, validation, and regulatory approval. Simple, interpretable evaluation strategies based on stable and recognizable image features may therefore provide a more reliable alternative in the surgical environment.

Taken together, previous studies underline the value of automated tissue analysis for brain tumor recognition and diagnosis. However, the application of label-free multiphoton technologies for real-time intraoperative imaging presents several challenges, including integration with other clinical or tactile information, interpretability of results for the surgeon, and regulatory constraints. Therefore, the application of simple evaluation strategies besides AI-based analysis might be more feasible. Hence, label-free multiphoton microscopy provides images of tissue morphochemistry that are very different from the stain- and antibody-based images usually used for diagnosis. For example, CARS and SRS typically address CH stretching vibrations and visualize lipids with high contrast. Lipids, however, might be washed out during alcohol-based tissue preparation so that lipid droplets (LD) appear as empty spaces in histology while being the most intense structures in CARS images (19). Therefore, identification of features suitable for intraoperative visual inspection of glioma is needed.

In this study, we focused on multiphoton techniques that have already been implemented for brain tumor visualization *in situ* (20, 21). We investigated the CARS, AF and SHG signals in glioma with the goal of identifying characteristic histological features in label-free images that could be recognized intraoperatively by the treating surgeon, thereby facilitating tumor identification and aiding in the assessment of tumor type.

## 2 Materials and methods

### 2.1 Samples

The study was approved by the ethics committee of the TU Dresden (EK 323122008), and written consent was obtained from the patients (all adult). Diagnosis was performed on separate tissue samples within the framework of clinical neuropathology.

Samples were obtained during routine surgery for treatment of glioma and stored at -80 °C. After embedding in cryomedium (Leica Biosystems Nussloch GmbH, Nussloch, Germany), cryosections of 16 µm (for multiphoton imaging) or 10 µm (for reference stainings) thickness were prepared and stored at -20 °C until further use.

### 2.2 Multiphoton microscopy

Tissue sections were allowed to thaw for 15 min at room temperature, rehydrated with phosphate buffered saline (PBS) and cover slipped. Imaging was performed as described elsewhere (6). Briefly, coherent anti Stokes Raman scattering (CARS), autofluorescence (AF), and second harmonic generation (SHG) were acquired using a multiphoton microscope (LSM 7 laser scanning module with Axio Examiner Z.1) through a W Plan Apochromat 20 ×/1.0 objective (all Carl Zeiss Microscopy GmbH, Jena, Germany). Femto Fiber pro NIR at 781 nm and TNIR at 1005 nm (both Toptica Photonics AG, Gräfelfing, Germany) having a pulse length of ~ 1 ps were used. Signal detection was realized using a bandpass filter 500–550 nm for autofluorescence (AF) and bandpass filters 633–647 nm and 381–399 nm for CARS (addressing the stretching vibration of C-H groups at 2850 cm^−1^) and SHG, respectively. For all acquisitions, the image pixel size was set to 0.27 µm and 2× line averaging was used. An area of 983 µm × 993 µm was imaged at several positions on each sample using a tiling procedure: 50 fields of view (400 × 800 pixel µm, corresponding to 106 µm × 213 µm) were acquired in a raster of 10 × 5 with 10% overlap.

To improve visualization, images were processed in Fiji (22). Stitching artifacts originating from uneven illumination of the field of view in the CARS channel were removed using the suppress stripes option of the FFT bandpass filter. Noise was removed by applying the Gaussion blur filter (radius of 0.5 or 1), and brightness and contrast were linearly adjusted.

### 2.3 Image analysis

Brain tumor related features in CARS-AF-SHG images were analyzed by visual inspection of the single channels. Lipid droplets (LD), cellular structures with cytoplasmic autofluorescence, and pathological blood vessels were identified and quantified on the CARS, AF or SHG signal, respectively. The localization of LD (extracellular or intracellular) was inferred from the combined CARS-AF-SHG images. The presence of features was rated by visual inspection in each field of view by a person blinded to the diagnosis of the case. The prevalence was calculated by determining the percentage of fields of view containing the feature of interest out of the total number of fields of view in a sample. Statistical analysis was performed using Prism 10.5.0 (GraphPad Software). Kruskal-Wallis test followed by Dunn’s multiple comparisons test was used to compare groups.

## 3 Results

Label-free multiphoton microscopy was performed on 102 tissue samples of human glioma (astrocytoma WHO2: n=9; astrocytoma WHO3: n=11; astrocytoma WHO4: n=8; oligodendroglioma WHO2: n = 17; oligodendroglioma WHO3: n = 4; glioblastoma: n=53) and allowed the visualization of tissue morphochemistry as reported previously (6). Several positions of approximately 1 × 1 mm were investigated for each sample.

### 3.1 Heterogeneity of tissue morphochemistry

**Figure 1:**
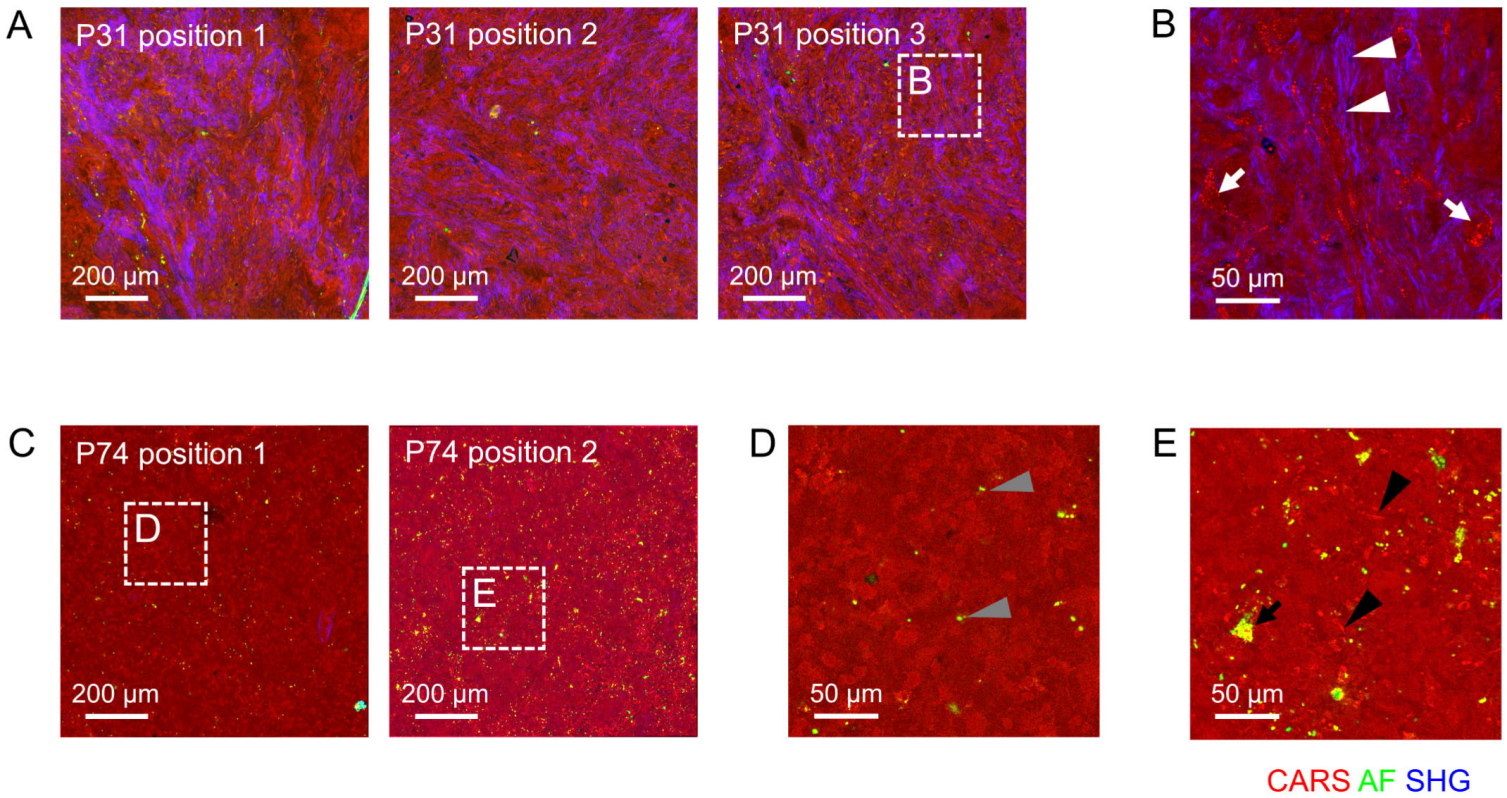
Multiphoton imaging of glioblastoma. **(A, C)** CARS-AF-SHG images of tissue morphochemistry of glioblastoma. Three **(A)** or two **(C)** different measurement positions are shown. **(B, D, E)** Zoom-in of the areas indicated in **(A, C)**. White arrows: intracellular lipid droplets, white arrowheads: collagen strands, gray arrowheads: punctuate autofluorescent structures, black arrows: cells with autofluorescent cytoplasm, black arrowheads: structures with intense CARS signal.

**Figure 1** shows examples of multiphoton images of two glioblastoma cases, the reference Ki67 immunohistochemistry is shown in **Supplementary Figure 1**. For the first case (P31), a strong SHG signal displaying fibrous structures was observed at all positions (**Figure 1A**), and the zoom-in reveals the presence of cells with intracellular LD (**Figure 1B**, white arrows), which are interspersed between the fibrous structures (**Figure 1B**, white arrowheads). For the second case (P74), the tissue structure appeared entirely different and almost no SHG signal was detected. The morphochemistry within one measurement position was again quite homogenous; however, profound differences were observed between position 1 and 2 (**Figure 1C**). Punctuate AF signals were found in position 1 (**Figure 1D**, gray arrowheads), while cells with intense AF and structures with intense CARS signal were found in position 2 (**Figure 1E**, black arrows and black arrowheads, respectively).

**Figure 2:**
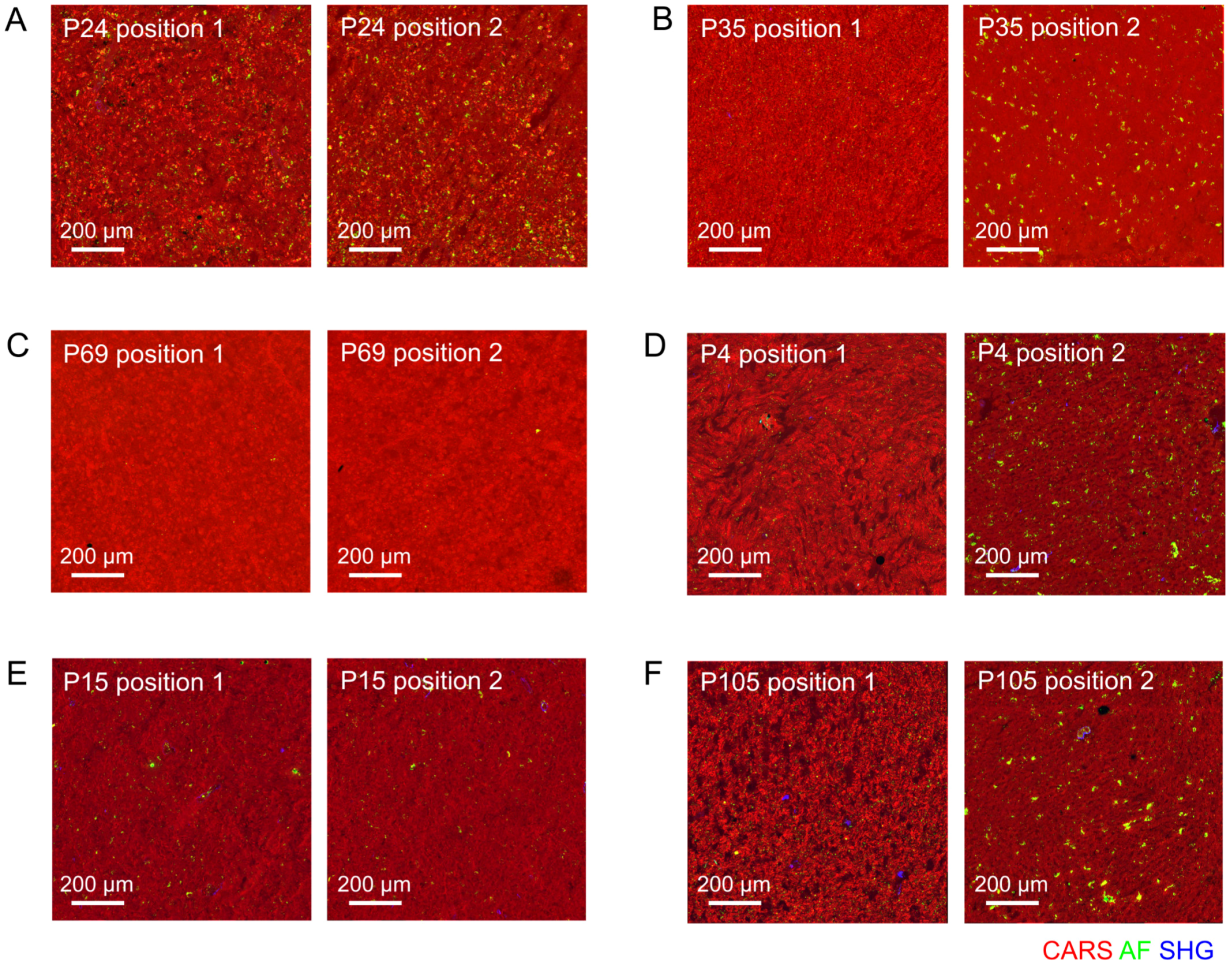
Multiphoton imaging of astrocytoma. CARS-AF-SHG images of two measurement positions are shown for each case. **(A, B)** Astrocytoma WHO 4. **(C, D)** Astrocytoma WHO 3. **(E, F)** Astrocytoma WHO 2.

**Figure 3:**
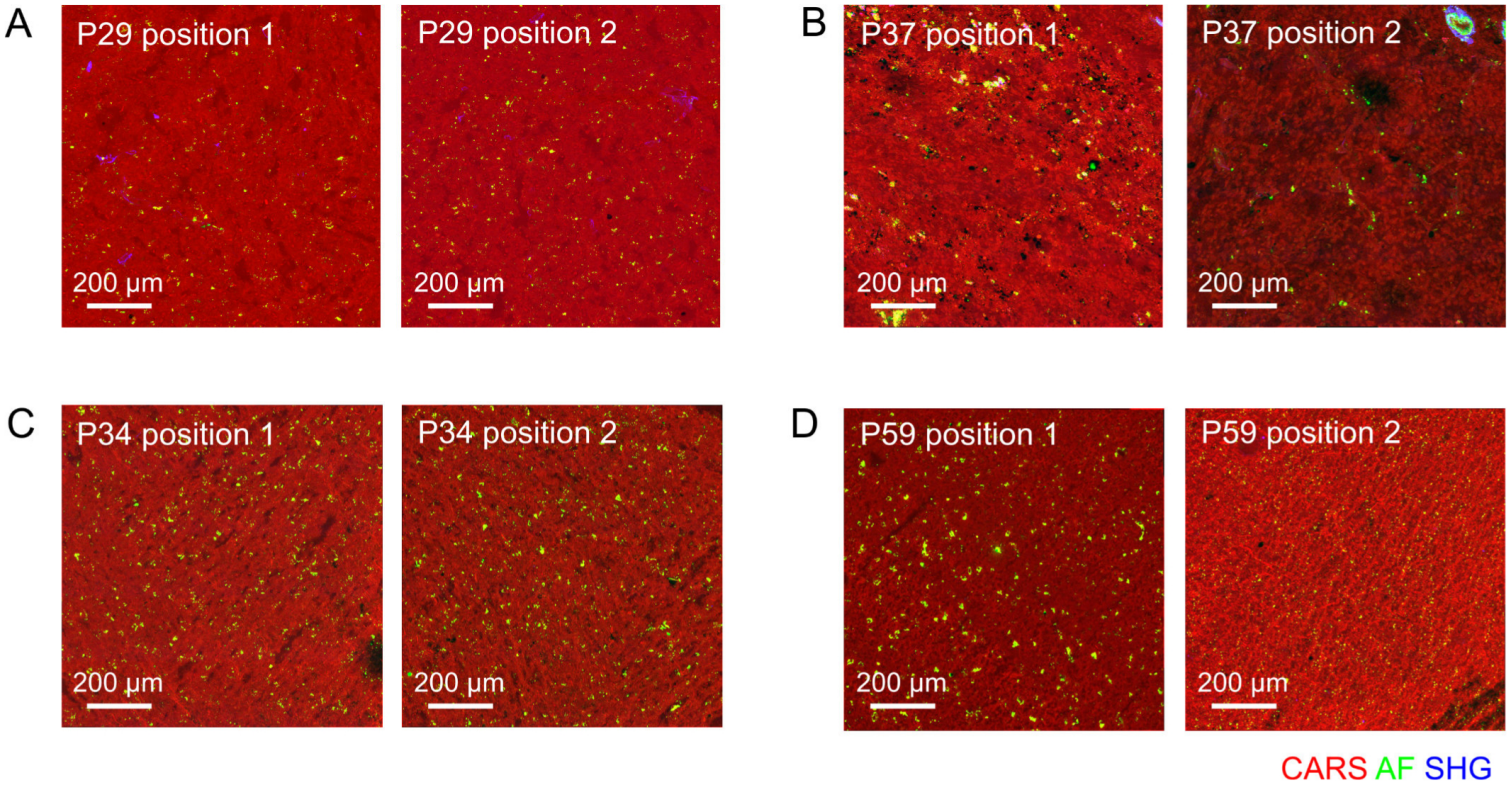
Multiphoton imaging of oligodendroglioma. CARS-AF-SHG images of tissue morphochemistry, two measurement positions are shown for each case. **(A, B)** Oligodendroglioma WHO 3. **(C, D)** Oligodendroglioma WHO 2.

The tissue morphochemistry of astrocytoma is shown in **Figure 2**. Similarly, for this type of glioma, the tissue appeared rather homogenous within one measurement position, while interpatient variability, as well as differences in homogeneity among the positions acquired on each sample, were observed. **Figure 2A** shows an example of an astrocytoma WHO 4, which has a similar appearance as the glioblastoma in **Figure 1C** at position 2. It shows punctuate AF and autofluorescent cells as well as small structures with intense CARS signal at both measurement positions. In contrast, the morphochemistry of astrocytoma WHO 4 shown in **Figure 2B** was heterogenous, with dense CARS signal and small punctuate AF structures at position 1 and autofluorescent cells at position 2. Likewise, we observed samples exhibiting either similar or heterogeneous morphochemistry at various measurement positions for astrocytoma WHO 3 (**Figures 2C, D**) and astrocytoma WHO 2 (**Figures 3E, F**). samples. Reference Ki67 immunohistochemistry of astrocytoma cases are shown in **Supplementary Figure 2**.

**Figure 3** shows examples of multiphoton imaging of oligodendroglioma and reference Ki67 immunohistochemistry is provided in **Supplementary Figure 3**. Again, the tissue morphochemistry was found to be homogenous at each measurement position. Among various positions, either a high degree of similarity or considerable differences were observed for oligodendroglioma WHO 3 (**Figures 3A, B**) or oligodendroglioma WHO 2 (**Figures 3C, D**).

**Figure 4:**
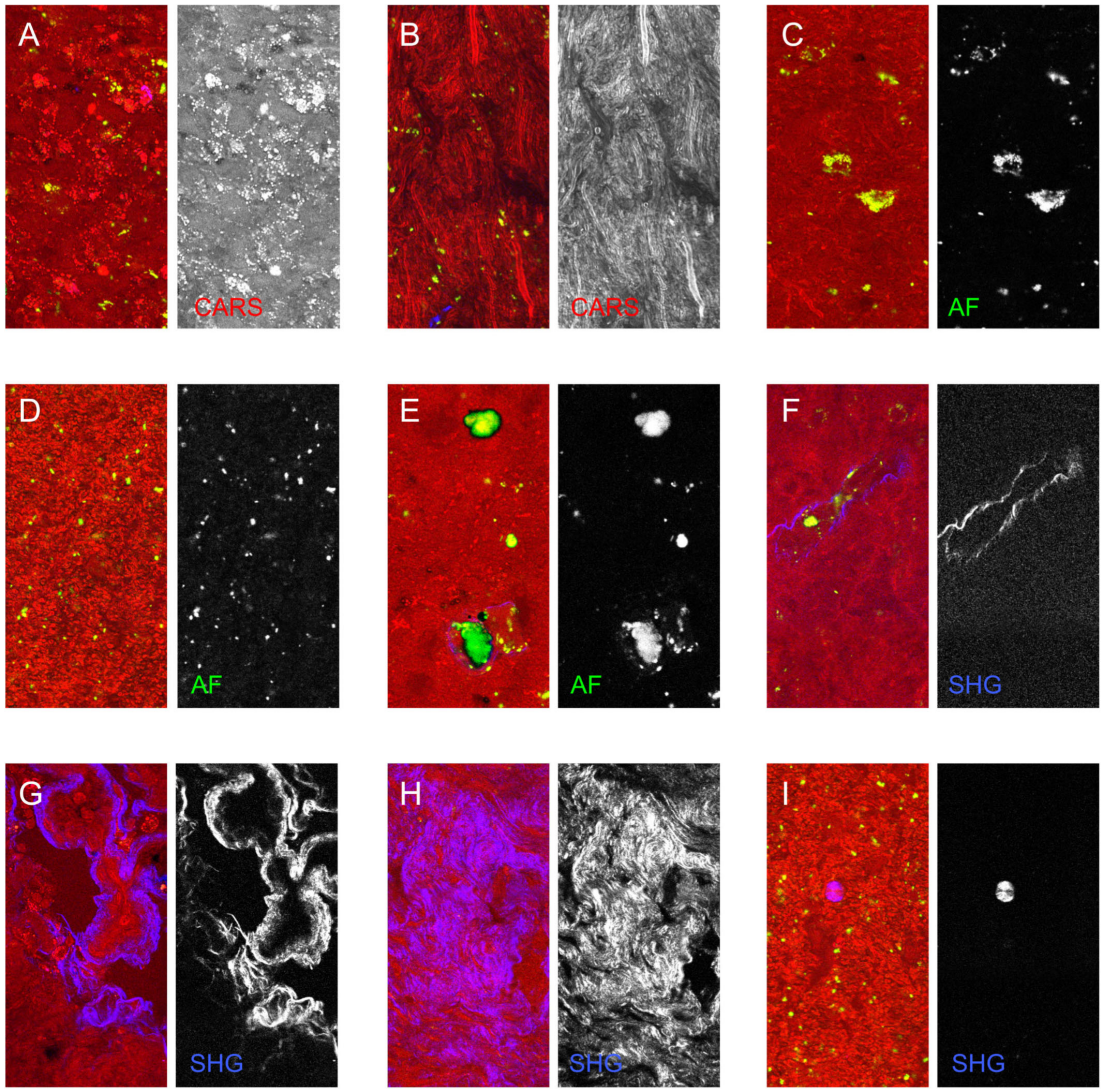
Examples of features in label-free multiphoton images of glioma (CARS: red; AF: green; SHG: blue). The overlay of all channels as well as the isolated channel of interest are shown. Images show one field of view (106 µm × 213 µm) **(A)** Lipid droplets visualized by CARS. **(B)** Axonal structures. **(C)** cells with cytoplasmic AF. **(D)** punctuate AF signal. **(E)** calcifications with intense AF. **(F–I)** tissue structures visualized by SHG-signal. **(F)** regular blood vessel **(G)** pathological vessel structure. **(H)** extracellular matrix remodeling. **(I)** corpora amylacea.

Taken together, approximately half of the glioma samples investigated (55/102) exhibited substantial heterogeneity of tissue morphochemistry among different measurement positions (glioblastoma: 31/53, astrocytoma WHO 4: 3/8, astrocytoma WHO 3: 5/11, astrocytoma WHO 2: 6/9, oligodendroglioma WHO 3: 2/4, oligodendroglioma WHO 2: 8/17). Moreover, the examples of the various glioma illustrate that the phenotype revealed in CARS-AF-SHG images is not directly related to a specific glioma subtype. However, there are several tissue components that can be reliably identified in the images based on previous research (6). CARS shows LD and axons (**Figures 4A, B**). Imaging of fluorescence reveals cells with cytoplasmic AF, punctuate AF and calcifications (**Figures 4C–E**). The SHG signal visualizes collagen of blood vessels and of extracellular matrix as well as corpora amylacea (12) (**Figures 4F–I**). Therefore, we analyzed the presence of features in the multiphoton images that may be related to tumor growth.

### 3.2 CARS signal

LD are a prominent hallmark of tumor related transformation of brain tissue and are visualized with high contrast using CARS microscopy tuned on CH-stretching vibrations (**Figure 4A**). Their localization can be further analyzed in combination with other modalities (6).

Intracellular LD were found in most glioma samples investigated (94/102). Samples without intracellular lipid droplets included mostly WHO grade 2 glioma (four oligodendroglioma cases and three astrocytoma cases), and one glioblastoma case. Extracellular LD were found in slightly fewer cases (83/102). As for the intracellular ones, most of the samples without extracellular LD were WHO grade 2 glioma (ten oligodendroglioma and three astrocytoma cases). Additionally, three samples of astrocytoma WHO 3 as well as three samples of glioblastoma were also lacking extracellular LD.

**Figure 5:**
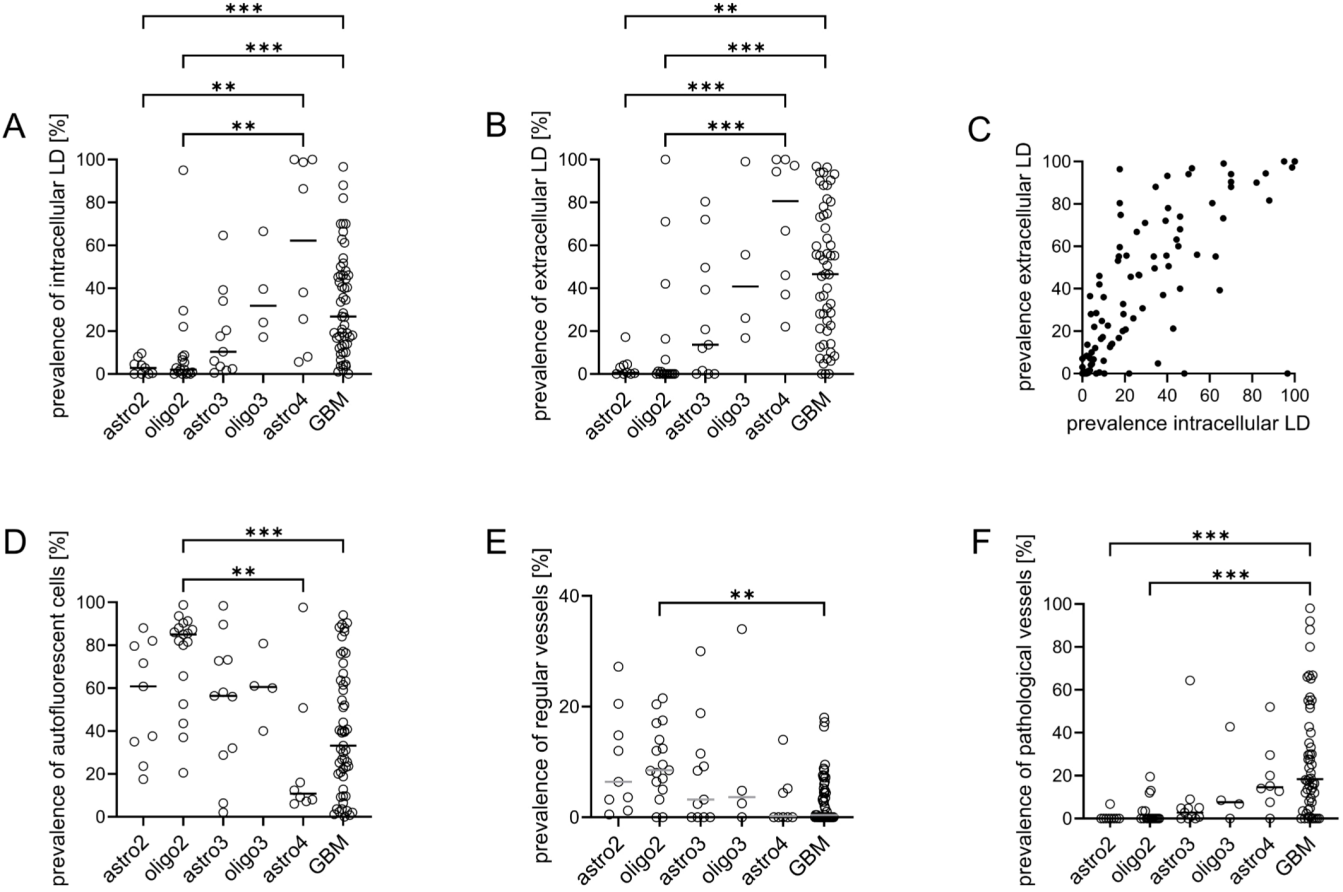
Features in CARS-AF-SHG images of glioma. **(A, B)** Prevalence of intracellular and extracellular lipid droplets (LD) based on CARS signal. **(C)** Correlation of intracellular and extracellular LD. **(D)** Prevalence of autofluorescent cells. **(E, F)** Prevalence of regular and pathological vessels based on SHG signal. Line indicates median, **P<0.01; ***P<0.001.

To further quantify the presence of LD with regard to future intraoperative applications, we investigated the presence of LD in each single field of view (size 106 µm × 213 µm). The prevalence was calculated as percentage of fields of view with LD on all fields of view of a sample. Generally, intra- and extracellular LD were observed less frequently in glioma with low WHO grade than in glioma with higher WHO grade. Significant differences were found for astrocytoma and oligodendroglioma WHO 2 versus astrocytoma WHO 4, as well as versus glioblastoma (**Figures A, B**). If intracellular droplets were detected, usually a similar or higher prevalence of extracellular lipid droplets was found (**Figure 5C**). The eight cases without intracellular lipid droplets did also have no (6/8) or very few extracellular droplets (2/8).

### 3.3 Autofluorescence signal

Cells with intense autofluorescence are found in gray matter and nuclear layers of non-neoplastic brain, while brain tissue as well as brain tumors might display punctuate AF (6, 8, 10). In this study, we observed cells with intense cytoplasmic AF (see **Figure 4C**) in in all samples except one glioblastoma. Analysis of the prevalence revealed that the occurrence of such cells is quite variable within each tumor type. Notably, a low prevalence under 10% was only found for astrocytoma WHO 3 (2/11), astrocytoma WHO 4 (4/8) and glioblastoma (12/53, **Figure 5D**). The median prevalence of autofluorescent cells was higher in oligodendroglioma WHO 2 and WHO 3 and astrocytoma WHO 2 and WHO 3 than in astrocytoma WHO 4 and glioblastoma, however, statistical differences were only found for oligodendroglioma WHO 2 versus astrocytoma WHO 4 and versus glioblastoma (**Figure 5D**). In addition to cells, calcifications within tumor tissue also have intense AF signals. In our study, we found those autofluorescent calcifications in only two samples (one oligodendroglioma WHO2, one glioblastoma, **Figure 4E**).

### 3.4 SHG signal

Fibrillar collagen can be visualized by SHG and, therefore, changes in the blood vessel network and changes in extracellular matrix (ECM) can be assessed by label-free multiphoton imaging. Regular vessels were characterized by fibrous structures with intense SHG signals around a lumen in longitudinal or cross-sectional views (**Figure 4F**). Vessels with a thickened wall, as indicated by SHG signal and an absence of lumina, as determined by inspection of CARS channel, were classified as pathological (**Figure 4G**). Regular blood vessels were found in most WHO 2 grade glioma (all astrocytoma; 15/17 oligodendroglioma), however, they were only found in approximately half of the astrocytoma WHO4 (3/8) and glioblastoma cases (28/53). They were usually observed sporadically only in a few fields of views, however in a few cases more frequently (in maximum in 34%). The prevalence of regular blood vessels was reduced in higher grade glioma and significant differences were observed between oligodendroglioma WHO2 and glioblastoma (**Figure 5E**). For pathological vessels, the opposite trend was observed. Those were only found in a few cases of WHO2 grade glioma (1/9 astrocytoma; 5/17 oligodendroglioma) and most astrocytoma WHO 4 (7/8) and glioblastoma (45/53). The median value indicated an increase of the prevalence of pathological vessels with malignancy of glioma, and significant differences were found for astrocytoma and oligodendroglioma WHO 2 versus glioblastoma, respectively (**Figure 5F**). In two samples, no SHG signal related to blood vessels was noticed. Moreover, ECM remodeling with extensive deposition of collagen (**Figure 4H**) was noted in 7/102 glioma samples, all of which were glioblastoma.

**Figure 6:**
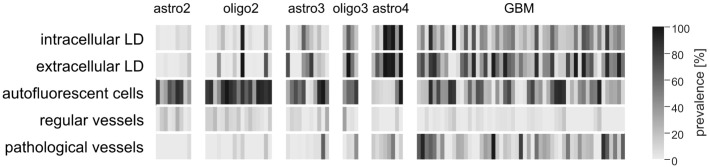
Presence of multiple features in label-free multiphoton images of glioma. Heatmap showing the prevalence of different features in CARS-AF-SHG images for each glioma case.

**Figure 6** visualizes the prevalence of intra- end extracellular LD, autofluorescent cells and regular and pathological vessels for each sample. It confirms that intracellular and extracellular lipid droplets usually occur within the same sample and that the prevalence of extracellular droplets is higher (compare **Figure 5C**). Additionally, a high prevalence of pathological vessels is found in cases without regular vessels. Considering the diagnosis, it becomes clear that WHO 2 glioma are characterized by lack of LD and presence of autofluorescent cells. Glioblastoma, on the other hand, are hallmarked by increased prevalence of pathological vessels and LD, while the presence of autofluorescent cells is reduced. However, glioblastoma morphochemistry is largely heterogenous.

The prevalence of multiple features found in CARS-AF-SHG images of tissue can be used for comprehensive characterization of each sample. **Supplementary Figure 4** shows several indices elaborated from different features combinations. Statistical analysis revealed significant differences among tumor types, multiple comparison of groups showed significant differences of index values of astrocytoma WHO 2 versus astrocytoma WHO 4 and glioblastoma as well as for oligodendroglioma WHO 2 versus astrocytoma WHO 4 and glioblastoma. However, there is a strong overlap among values for different tumor types, especially in cases in which the prevalence of autofluorescent cells is included in calculation of the index. This might be explained by the fact that those are predominantly found in gray matter and are thus related to tissue type in addition to their relevance for the detection of brain tumors.

### 3.5 Intraoperative scoring of CARS-AF-SHG images

Based on these findings, we developed a simple scoring scheme with regard to intraoperative use. Importantly, only a limited number of fields of view was included in the analysis, as tiling procedures are typically not implemented in endoscopes, which allow acquisition of single fields of view that are comparable with the one used in this investigation. Moreover, simplified features were defined to allow immediate image interpretation by the surgeon. We showed that if there are LD, both intra- and extracellular types are typically present (see **Figure 5C**). Therefore, the presence of LD was rated based on CARS signal without further specification of localization. A reduced prevalence of autofluorescent cells with increasing WHO grade was observed. However, as recognition of cells might be difficult in small fields of view and identification might be questionable in some cases, we additionally included the analysis of punctuate AF. Similarly, it is difficult to evaluate the structure of blood vessels in small image areas and it can also be difficult to separate these from changes of the ECM. Therefore, only the presence of SHG was evaluated as a criterion.

**Figure 7:**
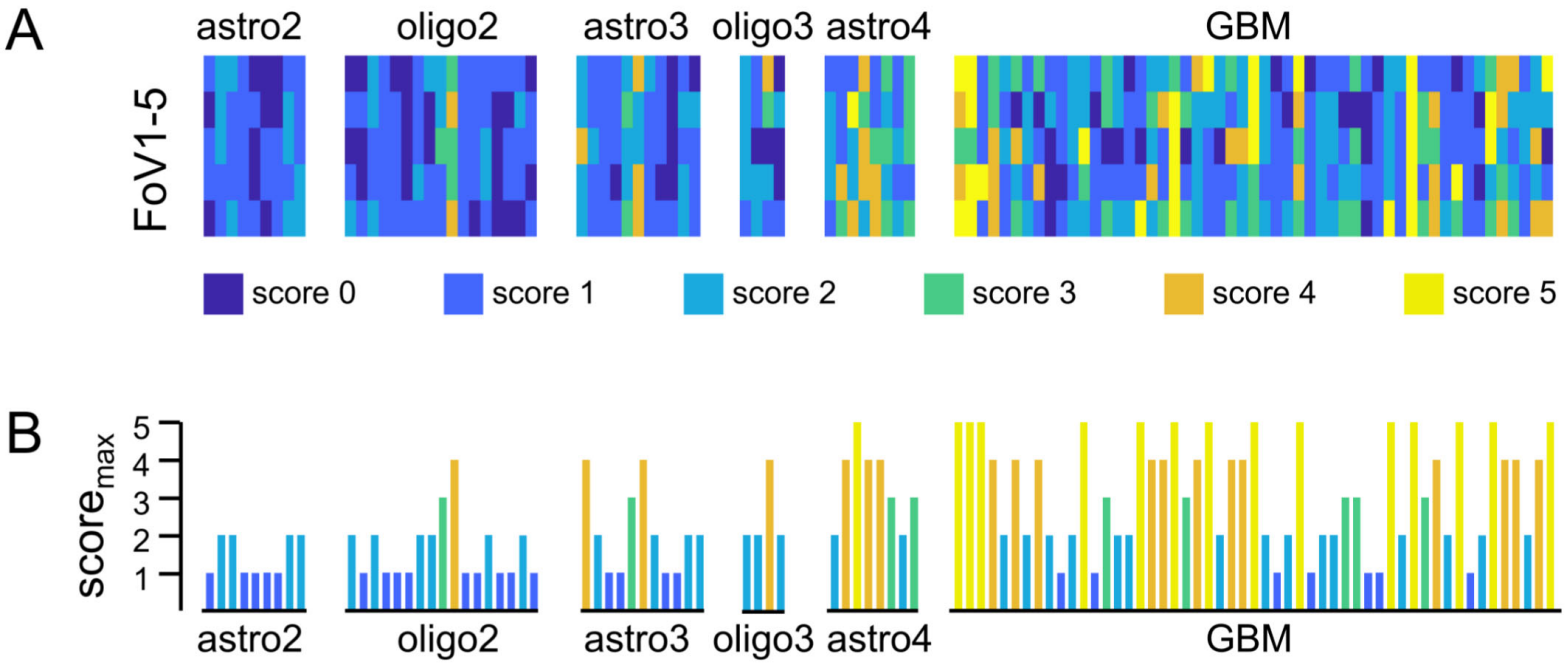
Score-based evaluation of CARS-AF-SHG data of glioma. The score was calculated based on presence of lipid droplets and SHG signal and lack of autofluorescent cells and punctuate AF signal **(A)** Score values for five fields of view (FoV) for each case. **(B)** Maximal score value (score_max_) found for each case. Cases are plotted in the same order as in **Figure 6**.

From these simplified features, a score for intraoperative use was calculated as follows: Presence of lipid droplets: 2 points, absence of autofluorescent cells: 1 point, absence of punctuate AF: 1 point, presence of SHG signal: 1 point. **Figure 7A** shows the resulting score in a color code for the five fields-of-view of all glioma samples investigated. Almost all astrocytoma WHO 2 and oligodendroglioma WHO 2 display scores between 0 and 2, while scores of 5 are only found in astrocytoma WHO 4 and glioblastoma. For astrocytoma WHO 2 and WHO 3, usually two different scores were found among the different fields of view. In accordance to the well-known high intratumoral heterogeneity, in half of the glioblastoma cases (26/52) three or more different scores were found among the five fields-of-view.

In the intraoperative setting, it will be necessary to obtain a conclusive rating for each case. In line with the paradigm that the histological diagnosis is formulated based on inspection of the most malignant area of the tumor, we suggest using the highest score (score_max_) found among the five fields of view. This approach also prevents low scores arising from imaging non-tumor areas or zones of low tumor infiltration from affecting and corroborating the final rating. **Figure 7B** shows that lower grade gliomas have mostly a lower score_max_ than higher grade gliomas. Specifically, all astrocytoma WHO 2 and most (15/17) oligodendroglioma WHO 2 cases received a score_max_ of 1 or 2. Interestingly, the oligodendroglioma WHO 2 case that was rated with a score_max_ of 4 demonstrated an atypical histopathology and underwent an extensive diagnostic process, where the possibility of a higher-grade glioma was discussed. For astrocytoma and oligodendroglioma, score_max_ of 1 to 4 are found. A score_max_ of 5 is found only for astrocytoma WHO 4 and glioblastoma cases.

## 4 Discussion

Label-free imaging allowed the comprehensive visualization of tissue morphochemistry of glioma and the integrated analysis of CARS, AF, and SHG confirmed substantial intra- and interpatient variability. In accordance with previous studies (6, 23), several tumor-related features were identified, including LDs, pathological blood vessels, altered ECM and reduction of AF signals. Our data indicates that these changes are associated with glioma malignancy.

Lipid droplets are well preserved in glioblastoma cryosections, even after PFA fixation, while methanol-acetone fixation is not compatible with CARS imaging of lipids, as lipids are washed away. (24). Likewise, paraffin embedding is not suited for CARS imaging, as external lipids are deposited in the tissue and the deparaffination includes washing with alcohol, thus largely affecting tissue lipids. The appearance of axonal myelin was similar in fresh brain tissue in comparison to unfixed cryosections (24). Moreover, CARS-AF-SHG imaging of human hippocampus identified the same tissue structures in fresh unfixed tissue samples as in tissue cryosections (8). In brain metastases of renal carcinoma, the technology detected intra- and extracellular lipid droplets, AF structures and SHG related to ECM remodeling. In this study, no qualitative differences were observed between fresh tissue and cryosections, although no quantitative analysis was performed (6). Also for glioblastoma, extra- as well as intracellular lipid droplets were found in fresh samples and the observed change in (extracellular) collagen visualized by SHG was similar (21). The comparison of *in situ* endoscopic imaging human brain tumors and analysis of tissue cryosections of the same brain tumor suggest that the pattern of tissue autofluorescence remains unchanged (10). However, the use of cryosections remains an approximation of the *in situ* situation and systematic studies are required to finally confirm that the features identified by label-free multiphoton microscopy do not change. Importantly, the suggested score neither requires differentiation between extra- and intracellular lipid droplets nor quantitative analysis of features. Therefore, it is particularly robust against minor changes of tissue features that could eventually occur after tissue resection. Here we showed that the presence of LD increases with glioma malignancy, a finding that is readily explained in the context of previous research. Lipid metabolism is substantially altered in cancer (25). LD are special organelles that fulfill essential roles in the context of cancer. Lipid storage in the form of intracellular LD is a hallmark of cancer cells and LD are involved in managing (oxidative) stress and regulating immunity (26). In the context of brain tumors, LDs are considered as an energy source that sustains cancer growth when extracellular nutrition supply is constrained and were suggested to promote tumor invasion (27). Moreover, cells of the tumor microenvironment might also display LD and a considerable proportion of intracellular LD observed in glioma might originate from lipid-laden macrophages (9, 28). Tumor-associated foam cells are a type of macrophages in human glioblastoma and are associated with worse prognosis and with hypoxia (9). Importantly those represent a potential therapeutic target.

Besides brain tumors, LDs in the brain are found in various inflammatory and demyelinating brain diseases, as well as in the stroke penumbra, and are associated with microglial activation (29, 30). In addition, oxidative and metabolic stress might also induce LD accumulation in astrocytes (31) and the changes in LD metabolism in astrocytes is associated with several neurological disorders, stroke, epilepsy, and glioma (32, 33). In the clinical setting, the differentiation of brain tumor (glioma) and other brain pathologies like stroke, or demyelinating lesions is an early step in the diagnostic workflow. Preoperative MRI plays a pivotal role in this process, offering high diagnostic accuracy and enabling clinicians to reliably identify glioma cases preoperatively. Consequently, the presence of LDs in other brain pathologies does not pose a practical limitation for glioma grading using intraoperative label-free imaging.

Cells and tissue contain several endogenous fluorophores including NADH, NADPH, FAD, aromatic amino acids, porphyrins, lipopigments, collagen and elastin which allow imaging of tissue structure and cell state and can be exploited for oncological imaging (34). The presence of autofluorescent cells is a prominent feature of gray matter of non-tumor brain (6) and different spectral AF signals were observed for gray matter, white matter and glioma (35). Pyramidal neurons of the human hippocampus were shown to display intense cytoplasmic AF (8). Therefore, the observed alterations in the AF pattern might be attributed to tumor growth and the subsequent displacement of normal brain tissue and neurons. However, cell-like structures with intense AF in glioma have been linked to the pattern of CD68-positve monocytes (36) and activated microglia showed intracellular AF, thus linking AF signals and the inflammatory response in the central nervous system (37). Thus, multiple cell types might contribute to the AF pattern in glioma. Interestingly, spectral patterns of brain metastases were shown to allow the identification of primary cancer site (38) underlining the importance of AF imaging for brain tumor diagnosis beyond glioma.

SHG imaging is widely accepted for visualization of pathology-induced tissue transformation (39). In normal human brain, almost no SHG signal is found with the exception of the weak collagen signal of blood vessels (40, 41). In brain tumors, vessel wall thickening as well as changes in ECM might lead to an intense SHG signal, making this modality a specific indicator of pathological tissue alterations (39). Collagen architecture was linked to survival in glioblastoma patients with atypical collagen being associated to shorter survival (40). However, changes in SHG signal of brain tumors are usually restricted to specific regions of the tumor (6, 42), thus the sensitivity of the approach is low. However, it is especially useful in combination with other modalities.

The prevalence of all described features was significantly different among low grade glioma (WHO 2) and most aggressive glioma types (WHO 4). However, significant differences among groups do not help in the diagnostic setting given the large range of values originating from the immense tumor heterogeneity among cases and within the same tumor. Besides being considered as an obstacle for precise and reliable diagnosis, intraoperative imaging would as well allow the assessment of tumor heterogeneity *in situ* and could allow regional tumor treatment as option. Here, the CARS imaging of LD, which are linked to tumor invasion and chemoresistance in glioblastoma (43), in combination with assessment of AF, which is an indicator of stem cell properties (44), could be of major interest.

Other tumor or malignancy related features might be extracted from the label-free images, like nuclear size (21, 23), however this is not feasible by visual inspection by the surgeon. As a limitation, the suggested clinical score does not provide straightforward diagnostic information for all cases. However, a score of five was unambiguously associated with astrocytoma WHO 4 and glioblastoma. Moreover, the surgeon has access to a multitude of additional clinical data like patient age, tumor localization, symptom onset, MRI data and tactile information, which is invaluable in the surgical context. All this information adds up to informed decision making with intraoperative imaging of tissue morphochemistry being one piece of evidence to be considered.

AI-based algorithms have demonstrated considerable potential for the reliable classification of CARS-AF-SHG images, exhibiting high levels of sensitivity and specificity in preclinical studies (7, 45, 46). However, most AI models are still trained on small, single-center datasets, which can lead to performance drift when applied to diverse patient populations. Moreover, deep learning models operate as a “black box” and results usually lack explainability. In contrast, tumor features in multiphoton images can be related to tumor properties, thereby providing mechanistic rational for clinical decision making. Last but not least, regulatory issues might impact the clinical implementation of AI based decision making.

Clinical management of glioma remains to face limitations and new treatment strategies are needed (47). When implemented with miniaturized multiphoton endoscopes (14, 45, 48) CARS-AF-SHG imaging holds strong potential for intraoperative delineation of brain tumors. Furthermore, the possibility of characterizing tissue types through direct visual inspection—without depending on AI-based algorithms—could support surgeons in real-time decision-making and contribute to more precise glioma management. Knowing the diagnosis of a brain tumor during surgery would have profound implications for neurosurgical decision making and patient management. First, it would directly influence the intended extent of resection. For tumors with diffuse infiltration patterns or known sensitivity to adjuvant therapies, a surgeon might prioritize functional preservation over aggressive resection, whereas for tumors in which maximal cytoreduction is strongly associated with improved outcome, a more extensive resection could be justified. Additional real-time diagnostics would therefore enable a more individualized balance between oncological benefit and neurological risk. Moreover, diagnostic information could enable the application of intraoperative therapies, tailored to specific tumor types. Particularly in the context of the substantial intratumoral heterogeneity, intraoperative analysis of morphochemistry provides a foundation for the development of innovative treatment concepts. This technology enables regional mapping of brain tumors with respect to relevant tumor characteristics. While it is undeniable that further research is needed in this area, intraoperative CARS-AF-SHG imaging could, in the future, allow for the differential assessment of multiple tumor regions prior to resection and lay the groundwork for the development of region-specific tumor therapies.

In conclusion, the proposed approach coupled with availability of suited endoscopic devices for intraoperative image acquisition, may extend the possibility of early diagnostics and contribute to accelerate postoperative decision making, reduce reliance on frozen sections, and streamline downstream molecular and therapeutic workflows.

## Data Availability

The datasets presented in this article are not readily available because of ethical reasons. Requests to access the datasets should be directed to ortrud.uckermann@ukdd.de.
