# Supplementary material for: Label-free multiphoton microscopy for intraoperative identification of glioma features and tumor heterogeneity
Source: Front Oncol. 2026 Jun 22;16:1802324. doi: 10.3389/fonc.2026.1802324 (PMC13333430; doi:10.3389/fonc.2026.1802324)
Supplement: Supplementary file 1 [file DataSheet1.pdf]

Supplementary Material

Label-Free Multiphoton Microscopy for intraoperative Identification of Glioma Features and Tumor Heterogeneity

1 Supplementary Data:

Ki67 Histology: The sections were fixed in methanol-acetone solution at -20 °C, and then dried for 30 minutes. For heat-induced epitope recovery, cryosections were incubated in citrate buffer (pH=6.0) for 40 min in a steamer. Peroxidase blocking was performed with 0.3% hydrogen peroxide-methanol solution for 15 min, followed by incubation in block serum (0.3% Triton X-100 and 1% bovine serum albumin in PBS) for 1 hour. The primary antibody (Novocastra monoclonal mouse antibody Ki67 antigen, Leica Biosystems) was added to the blocking serum (1:200), and the sections were incubated over night at 4C. After washing in PBS, the sections were incubated with the secondary antibody (Histofine Simple Stain MAX PO (M), Nichirei Biosciences), followed by colorimetric detection of the antibody signal (HistoGreen Kit, Linaris). The samples were washed again in PBS and counterstained with nuclear red. After dehydration in ethanol and xylene, the sections were mounted with Entellan.

2 Supplementary Figures and Tables

2.1 Supplementary Figures

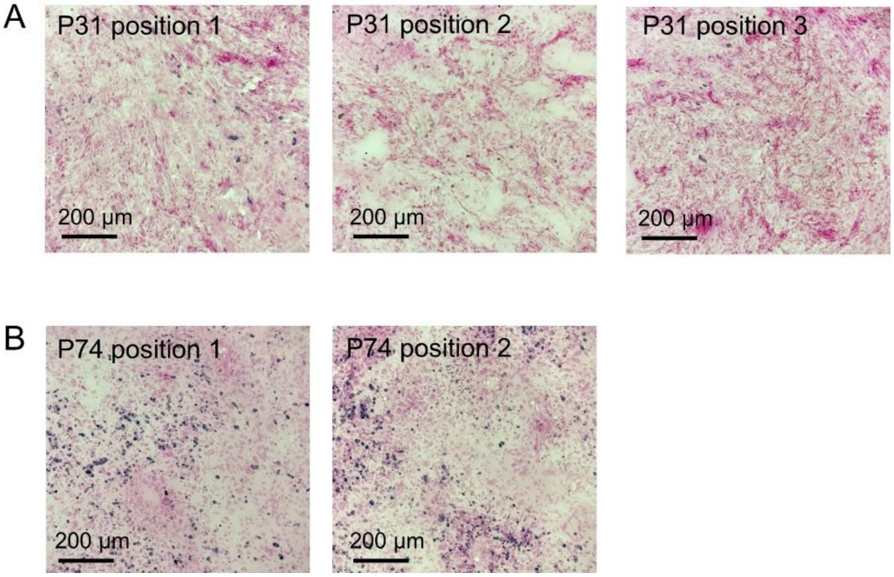

Supplementary Figure S1. Ki67 reference staining of the glioblastoma samples shown in Figure 1, Three (A) or two (B) different measurement positions are shown. Ki67 detection: histogreen; counterstaining: nuclear fast red.

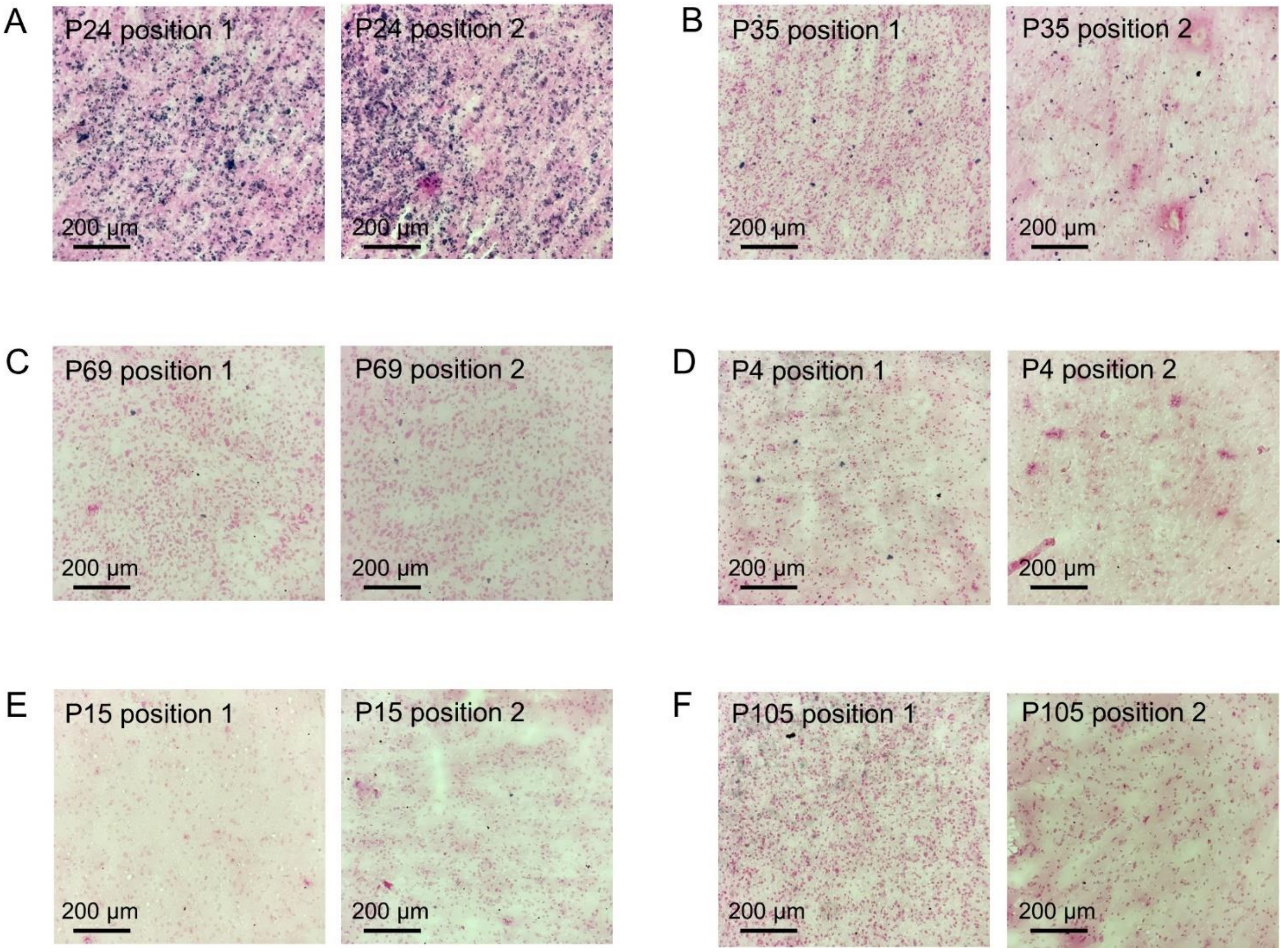

Supplementary Figure S2. Ki67 reference staining of the samples shown in Figure 2, two measurement positions are shown for each case. Ki67 detection: histogreen; counterstaining: nuclear fast red. **A/B:** Astrocytoma WHO 4. **C/D:** Astrocytoma WHO 3. **E/F:** Astrocytoma WHO 2.

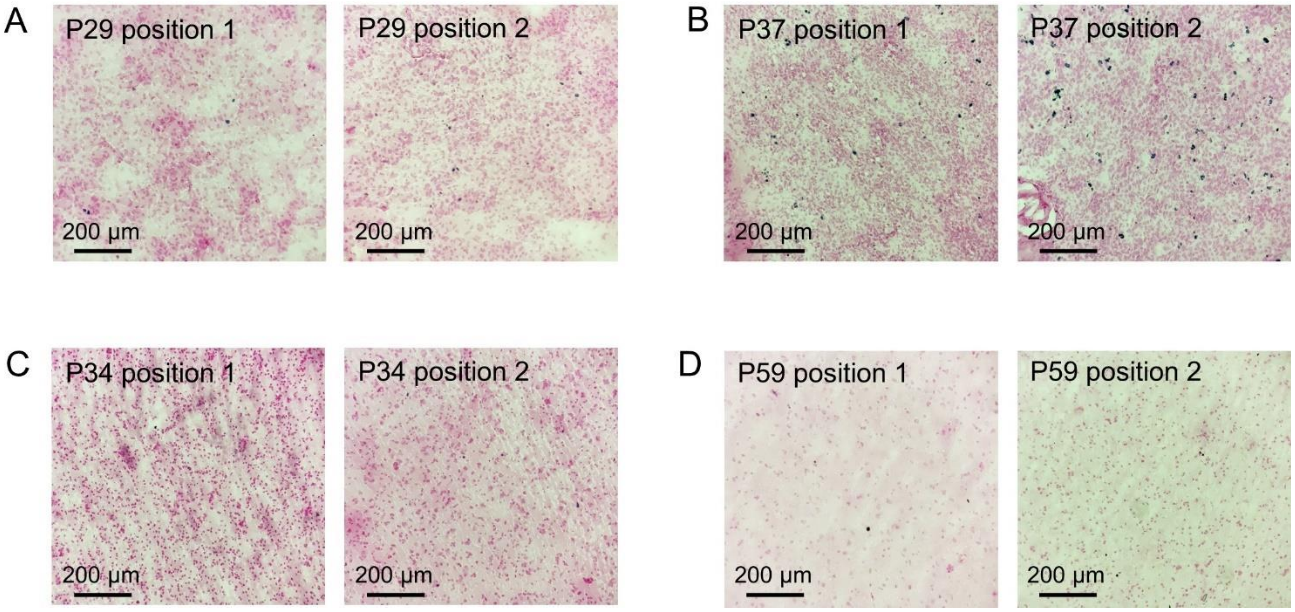

Supplementary Figure S3. Ki67 reference staining of the samples shown in Figure 3, two measurement positions are shown for each case. Ki67 detection: histogreen; counterstaining: nuclear fast red. **A/B:** Oligodendroglioma WHO 3. **C/D:** Oligodendroglioma WHO 2.

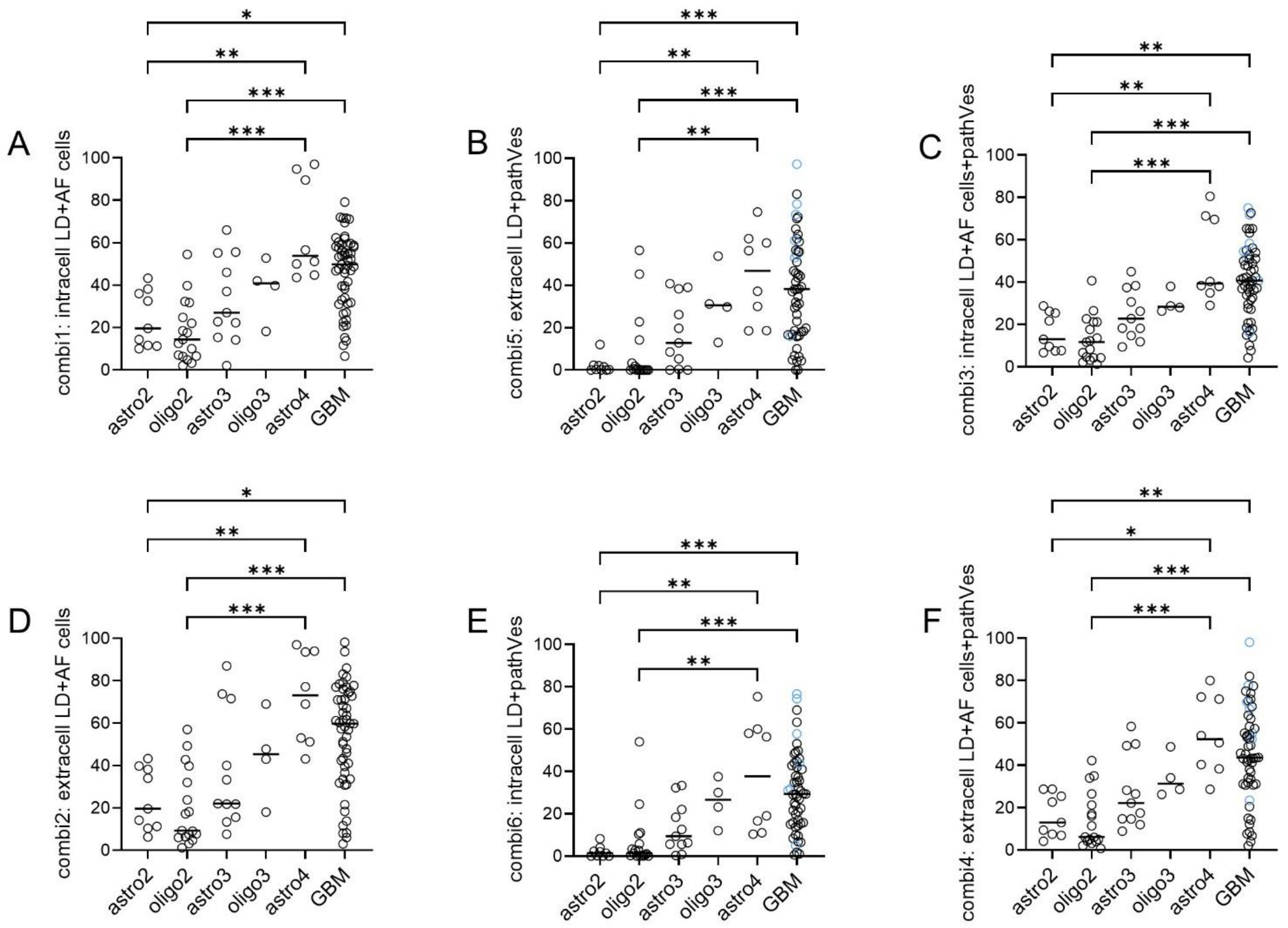

Supplementary Figure 4. Indices calculated for different combination of features for each sample.
